# Does “transylvania effect” oversway emergent psychiatric presentations?: a quantitative study from Turkey

**DOI:** 10.1192/j.eurpsy.2026.10934

**Published:** 2026-07-31

**Authors:** O. Gokcen, C. Yilmaz, N. Tig

**Affiliations:** 1 Psychiatry, Kutahya Health Science University, Kutahya, Türkiye; 2 Psychiatry, Sligo Leitrim Mental Health Services, Sligo, Ireland; 3 Kutahya Health Science University, Kutahya, Türkiye

## Abstract

**Introduction:**

The *Transylvania effect* refers to a concept that suggests a correlation between the lunar cycle and human behavior. Within this framework, violent behavior, suicide attempts, criminal activity, injuries, and accidents are the most prominent circumstances that have been investigated, with claims that such events are exacerbated during particular lunar phases (Gokhale and Kumar, *Cureus*, 2023; 15[11]). A lunar month lasts approximately 29.5 days and progresses through eight distinct synodic phases: new moon, waxing crescent, first quarter, waxing gibbous, full moon, waning gibbous, last quarter, and waning crescent.

**Objectives:**

This study aims to examine whether psychiatric emergencies vary according to lunar phases. To address this objective, psychiatric presentations were categorized into five groups. The specific aims were: 1)To determine the annual number of psychiatric presentations to the emergency department over an eight-year period 2) To classify the total number of presentations according to the eight synodic phases 3) To investigate potential associations between psychiatric diagnostic groups and lunar phases.

**Methods:**

A total of 4,488 patients who presented to the Emergency Department of Kütahya Health Sciences University with psychiatric complaints between January 1, 2016, and October 24, 2024, were retrospectively evaluated in relation to the lunar phase at the time of presentation. Eligible patients were categorized into the following groups: suicide attempts, psychotic or manic episodes, anxiety or panic attacks, alcohol/substance withdrawal or intoxication, and other psychiatric conditions. Statistical analyses were performed using Poisson regression and negative binomial regression.

**Results:**

An increase was observed in suicide attempts, psychotic-manic episodes, anxiety-panic attacks, and total psychiatric emergency visits during the new moon and full moon phases. Regression analyses were conducted to confirm statistical significance. For this analysis, the new moon and full moon phases were compared against the remaining lunar phases. These remaining phases were grouped into a four-phase model consisting of new moon, waxing moon, full moon, and waning moon. Compared to the waning moon phase, the total number of psychiatric emergency presentations was significantly higher during both the full moon and new moon phases (p < 0.001 for each).

**Image 1:**
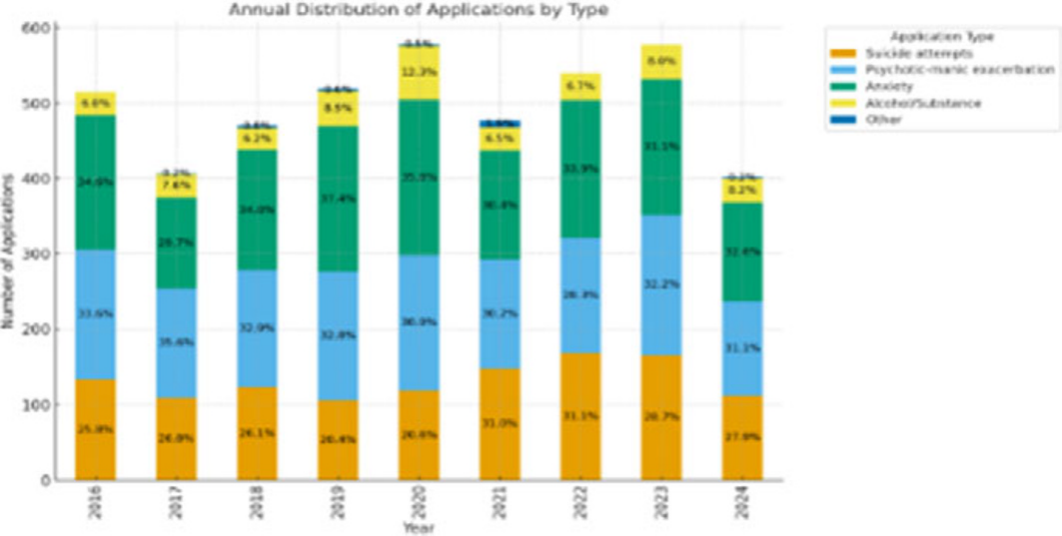
Image 1: Long description.

**Image 2:**
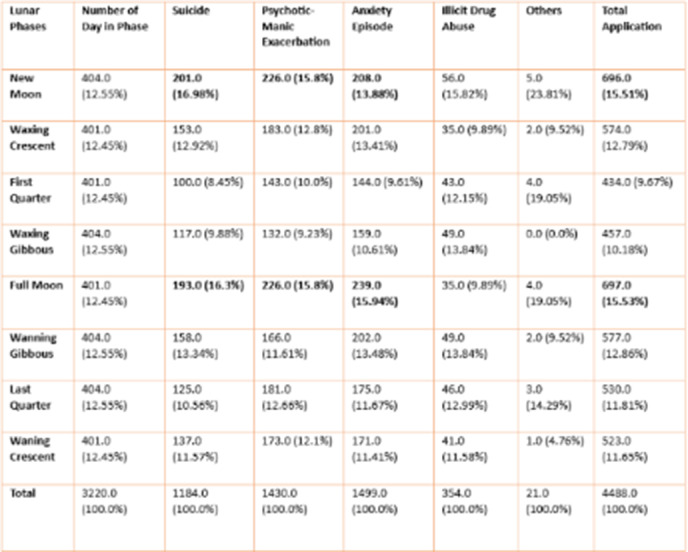
Image 2: Long description.

**Image 3:**
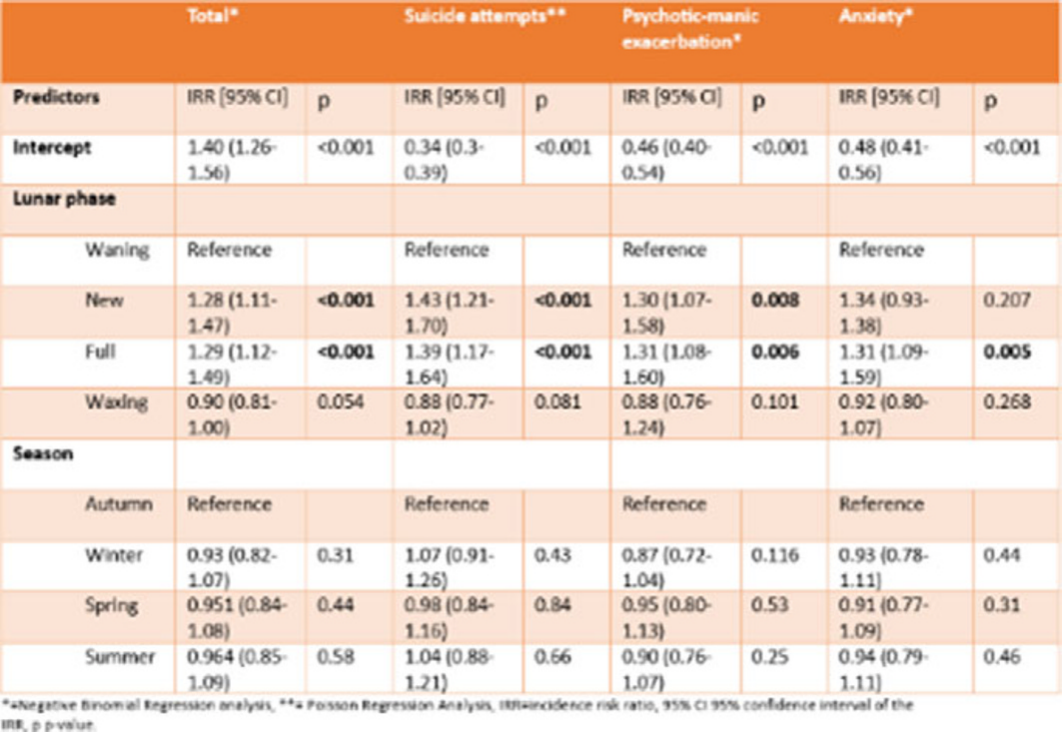
Image 3: Long description.

**Conclusions:**

The *Transylvania effect*, often regarded as a folkloric or mystical phenomenon, appears to influence psychiatric emergency presentations in this study. The new moon and full moon phases were associated with significant increases in total psychiatric emergencies, suicide attempts, and psychotic-manic exacerbations. Although much of the current literature disputes the existence of the *Transylvania effect*, these findings, derived from a large sample size, provide evidence that challenges prevailing skepticism.

**Disclosure of Interest:**

None Declared

## Long descriptions

### Image 1: Long description

The Y-axis is labeled Number of Applications, ranging from 0 to 600. The X-axis is labeled Year, with bars for 2016 through 2024. A legend on the right identifies five categories: Suicide attempts (orange), Psychotic-manic exacerbation (light blue), Anxiety (green), Alcohol/Substance (yellow), and Other (dark blue).

Each bar represents the total applications for that year, with internal segments labeled by percentage:

* 2016: Total ~510. Suicide attempts 25.9 percent, Psychotic 33.6 percent, Anxiety 34.9 percent, Alcohol 5.6 percent.

* 2017: Total ~410. Suicide attempts 26.9 percent, Psychotic 35.6 percent, Anxiety 29.7 percent, Alcohol 7.6 percent.

* 2018: Total ~470. Suicide attempts 26.1 percent, Psychotic 32.9 percent, Anxiety 34.0 percent, Alcohol 6.2 percent.

* 2019: Total ~520. Suicide attempts 20.4 percent, Psychotic 32.8 percent, Anxiety 37.4 percent, Alcohol 8.5 percent.

* 2020: Total ~580 (peak). Suicide attempts 20.6 percent, Psychotic 30.9 percent, Anxiety 35.9 percent, Alcohol 12.3 percent.

* 2021: Total ~480. Suicide attempts 31.0 percent, Psychotic 30.2 percent, Anxiety 30.4 percent, Alcohol 6.5 percent.

* 2022: Total ~540. Suicide attempts 31.1 percent, Psychotic 28.3 percent, Anxiety 33.9 percent, Alcohol 6.7 percent.

* 2023: Total ~570. Suicide attempts 28.7 percent, Psychotic 32.2 percent, Anxiety 31.1 percent, Alcohol 8.0 percent.

* 2024: Total ~400. Suicide attempts 27.9 percent, Psychotic 31.1 percent, Anxiety 32.6 percent, Alcohol 8.2 percent.

The Other category remains below 1 percent across all years, appearing as a thin dark blue line at the top of each bar.

Navigate back to Image 1:.

### Image 2: Long description

The table consists of 9 rows and 8 columns.

Column headers from left to right: Lunar Phases, Number of Day in Phase, Suicide, Psychotic-Manic Exacerbation, Anxiety Episode, Illicit Drug Abuse, Others, and Total Application.

Row data by Lunar Phase:

* New Moon: 404.0 days (12.55%), 201.0 suicides (16.98%), 226.0 psychotic (15.8%), 208.0 anxiety (13.88%), 56.0 drug abuse (15.82%), 5.0 others (23.81%), 696.0 total (15.51%).

* Waxing Crescent: 401.0 days (12.45%), 153.0 suicides (12.92%), 183.0 psychotic (12.8%), 201.0 anxiety (13.41%), 35.0 drug abuse (9.89%), 2.0 others (9.52%), 574.0 total (12.79%).

* First Quarter: 401.0 days (12.45%), 100.0 suicides (8.45%), 143.0 psychotic (10.0%), 144.0 anxiety (9.61%), 43.0 drug abuse (12.15%), 4.0 others (19.05%), 434.0 total (9.67%).

* Waxing Gibbous: 404.0 days (12.55%), 117.0 suicides (9.88%), 132.0 psychotic (9.23%), 159.0 anxiety (10.61%), 49.0 drug abuse (13.84%), 0.0 others (0.0%), 457.0 total (10.18%).

* Full Moon: 401.0 days (12.45%), 193.0 suicides (16.3%), 226.0 psychotic (15.8%), 239.0 anxiety (15.94%), 35.0 drug abuse (9.89%), 4.0 others (19.05%), 697.0 total (15.53%).

* Waning Gibbous: 404.0 days (12.55%), 158.0 suicides (13.34%), 166.0 psychotic (11.61%), 202.0 anxiety (13.48%), 49.0 drug abuse (13.84%), 2.0 others (9.52%), 577.0 total (12.86%).

* Last Quarter: 404.0 days (12.55%), 125.0 suicides (10.56%), 181.0 psychotic (12.66%), 175.0 anxiety (11.67%), 46.0 drug abuse (12.99%), 3.0 others (14.29%), 530.0 total (11.81%).

* Waning Crescent: 401.0 days (12.45%), 137.0 suicides (11.57%), 173.0 psychotic (12.1%), 171.0 anxiety (11.41%), 41.0 drug abuse (11.58%), 1.0 others (4.76%), 523.0 total (11.65%).

Bottom Row (Total): 3220.0 days, 1184.0 suicides, 1430.0 psychotic, 1499.0 anxiety, 354.0 drug abuse, 21.0 others, 4488.0 total applications. All total percentages are 100.0%.

Navigate back to Image 2:.

### Image 3: Long description

A data table with nine columns. The first column lists Predictors, followed by four pairs of columns for Total, Suicide attempts, Psychotic-manic exacerbation, and Anxiety. Each pair includes I R R [95% C I] and p-value.

Predictors and results:

* Intercept: Total I R R 1.40, Suicide 0.34, Psychotic 0.46, Anxiety 0.48. All p < 0.001.

* Lunar phase (Waning is the reference):

- New: Total I R R 1.28 (p < 0.001), Suicide 1.43 (p < 0.001), Psychotic 1.30 (p 0.008), Anxiety 1.34 (p 0.207).

- Full: Total I R R 1.29 (p < 0.001), Suicide 1.39 (p < 0.001), Psychotic 1.31 (p 0.006), Anxiety 1.31 (p 0.005).

- Waxing: Total I R R 0.90 (p 0.054), Suicide 0.88 (p 0.081), Psychotic 0.88 (p 0.101), Anxiety 0.92 (p 0.268).

* Season (Autumn is the reference):

- Winter: Total I R R 0.93, Suicide 1.07, Psychotic 0.87, Anxiety 0.93.

- Spring: Total I R R 0.951, Suicide 0.98, Psychotic 0.95, Anxiety 0.91.

- Summer: Total I R R 0.964, Suicide 1.04, Psychotic 0.90, Anxiety 0.94.

Footnotes indicate Negative Binomial Regression for Total, Anxiety, and Psychotic-manic exacerbation, and Poisson Regression for Suicide attempts. I R R stands for incidence risk ratio and C I stands for confidence interval.

Navigate back to Image 3:.

